# ZFP57 suppress proliferation of breast cancer cells through down-regulation of MEST-mediated Wnt/β-catenin signalling pathway

**DOI:** 10.1038/s41419-019-1335-5

**Published:** 2019-02-20

**Authors:** Lie Chen, Xiaowei Wu, Hui Xie, Na Yao, Yiqin Xia, Ge Ma, Mengjia Qian, Han Ge, Yangyang Cui, Yue Huang, Shui Wang, Mingjie Zheng

**Affiliations:** Department of Breast Surgery, The First Affiliated Hospital of Nanjing Medical University, 300 Guangzhou Road, Nanjing, Jiangsu 210029 China

## Abstract

Activation of oncogenes by promoter hypomethylation plays an important role in tumorigenesis. Zinc finger protein 57 (ZFP57), a member of KRAB-ZFPs, could maintain DNA methylation in embryonic stem cells (ESCs), although its role and underlying mechanisms in breast cancer are not well understood. In this study, we found that ZFP57 had low expression in breast cancer, and overexpression of ZFP57 could inhibit the proliferation of breast cancer cells by inhibiting the Wnt/β-catenin pathway. MEST was validated as the direct target gene of ZFP57 and MEST may be down-regulated by ZFP57 through conserving DNA methylation. Furthermore, overexpression of MEST could restore the tumour-suppressed and the Wnt/β-catenin pathway inactivated effects of ZFP57. ZFP57-MEST and the Wnt/β-catenin pathway axis are involved in breast tumorigenesis, which may represent a potential diagnostic biomarker, and provide a new insight into a novel therapeutic strategy for breast cancer patients.

## Introduction

Embryonic stem cells (ESCs), a type of cell isolated from early embryos or primitive gonads, are characterised by unlimited proliferation, self-renewal and multi-directional differentiation ability^1,2^. Self-renewal and differentiation capacity, the hallmark traits of stem cells, are mirrored by the high-proliferative capacity and phenotypic plasticity of tumour cells^3^. Previous research studies have clarified that networks of diverse transcription factors stimulate the expression of a series of genes, which could preserve self-renewal in ESCs^4–6^. Recently, it has been argued that the Wnt/β-catenin pathway^7–10^, STAT3 pathway^11–13^ and Hedgehog^14^ signalling pathways, which are involved in regulating ESCs’ cellular progression, could play critical roles in tumour initiation and development. Briefly, the regulators and signal transduction pathways involved in ESCs’ self-renewal may play critical roles in cancer cell proliferation and growth in the same way.

ZFP57, a member of the KRAB zinc finger family of proteins (KRAB-ZFPs), is an ES-specific transcription factor. It has been reported that ZFP57 can bind to its co-factor, such as KRAB-associated protein 1 (KAP1), through the KRAB domain, and then participate in genome imprinting, by maintaining DNA methylation in ESCs^15–18^. Previous results have shown that ZFP57 was able to regulate the DNA methylation level through interacting with DNA methyltransferase (DNMT) 1, 3A, and 3B in ESCs^19^. On the other hand, abnormal DNA methylation is an important phenomenon in tumorigenesis^20,21^. Due to aberrant methylation in CpG islands, the expression of tumour suppressor genes (TSGs) or oncogenes has altered significantly in certain kinds of cancers^22,23^. Moreover, DNA methylation may be one of the earliest, most robust, and frequent changes in cancer development^24,25^.

As is known, ZFPs are the largest transcription factor family members in mammals, one third of which are KRAB-ZFPs^19^. KRAB-ZFPs could inhibit or promote carcinogenesis. Zhang et al. found that ZNF382 could suppress tumour cell proliferation and promote apoptosis in oesophageal squamous cell carcinoma^26^. ZFP545 was also described as a tumour suppressor in multiple types of tumours, including breast cancer^27,28^. These studies also clarified that both ZNF382 and ZFP545 could suppress tumours by inhibiting the Wnt/β-catenin pathway^26^. However, little is known about the expression and function of ZFP57 in breast cancer.

In this study, we investigated the expression levels of ZFP57 and its biological functions in breast cancer. To verify our hypothesis that ZFP57 can participate in tumorigenesis through regulating DNA methylation of TSGs or oncogenes in breast cancer, we further explored the mechanism of ZFP57 on tumour suppression of breast cancer.

## Materials and methods

### Cell lines and cell culture

SUM1315 cell line was provided by Stephen Ethier at the University of Michigan. HBL-100 cell line was sourced from the cell bank of the Shanghai Institute of Biological Sciences, and the Chinese Academy of Sciences. All other cell lines were obtained from the American Tissue Culture Collection (ATCC), including MCF-7, ZR-75-1, T47D, and MDA-MB-231. SUM1315, MCF-7, and MDA-MB-231 cells were grown in Dulbecco’s modified eagle’s medium (DMEM) (Gbico, Detroit, MI, USA) with 10% foetal bovine serum (FBS) (Gbico, Detroit, MI, USA) and antibiotics (1% penicillin/streptomycin, Gibco, Detroit, MI, USA). In addition to this, the HBL-100, ZR-75-1 and T47D cells were grown in RPMI1640 (Gbico, Detroit, MI, USA) with 10% FBS and antibiotics. All of these cells were cultured at 37 ^o^C, in a humidified atmosphere of 95% air and 5% CO_2._

### Tissue specimens

Breast cancer tissues and their paired adjacent normal tissues (80 pairs) were collected from patients of the First Affiliated Hospital of Nanjing Medical University (Nanjing, China). None of these patients received any preoperative treatment. After surgical removal, cryopreservation of all tissues was maintained at −80 °C until use. Before the collection of specimens, all patients or their relatives gave informed consent, and specimens utilised in this study were approved by the Institutional Ethical Board of the First Affiliated Hospital of Nanjing Medical University (Nanjing, China).

### Lentivirus transfection

The ZFP57 overexpression/knock-down lentivirus, and negative controls (LV-NC/sh-Ctrl), were all purchased from GenePharma (Shanghai, China). Cells (SUM1315) were transfected with LV-ZFP57 and LV-NC at 40–50% confluence. In addition to this, cells (MCF-7) were transfected with sh-ZFP57 and sh-Ctrl. Stable pooled populations of breast cancer cells were selected by using 3 μg/ml puromycin (VWR, USA) for 10−15 days.

### Plasmid and small interfering RNA (siRNA) transfection

According to the manufacturer’s instructions, Lipofectamine 3000 (Invitrogen, Carlsbad, CA, USA) should be used to transfect plasmids and siRNAs of MEST (GenePharma) into breast cancer cells.

### Quantitative real-time PCR

Total RNA was extracted from tissues and cells using RNAiso Plus (Takara, Kusatsu, Japan). After determining RNA concentration, the cDNA was synthesised using a reverse transcription kit (Takara). A SYBR Green Master Mix Kit (Roche, Reinach, Switzerland) was utilised to perform the quantitative real-time polymerase chain reaction (qRT-PCR) in a 7500 Real-time PCR System (Applied Biosystems, Foster City, CA, USA), and the expression of target mRNAs was normalised based on GAPDH. Melting curves were used to monitor nonspecific amplifications. The relative expression level was calculated using the 2^–^^ΔΔCt^ method. To verify the results, we performed each PCR amplification in triplicate.

The primers for the target genes in the study are listed in Supplementary file 1 (Part.1).

### Western blot

Cells were harvested for Western blot analysis as described^29^.

The anti-ZFP57 (abcam, Cambridge, UK, 1:1000), the anti-MEST (abcam, 1:2000), the anti-β-catenin (abcam, 1:5000), the anti-cyclin D1 (abcam, 1:5000), the anti-c-Myc (abcam, 1:5000), the anti-GAPDH (CellSignaling, Danvers, MA, 1:1000) and the anti-α-Tubulin (abcam, 1:5000) were used as the primary antibodies. GAPDH and α-Tubulin were used as an internal control. A 1:5000–10,000 dilution of the Peroxidase-conjugated Affinipure GOAT anti-Mouse and anti-Rabbit IgG (H + L) (Jackson Immunoresearch, USA) was used as the secondary antibody.

### Chromatin immunoprecipitation assay

Chromatin immunoprecipitation (ChIP) analysis was performed using ChIP kits (17–371, EZ-ChIP, Millipore, Bedford, MA, USA). Briefly, SUM1315 and MCF-7 cells transfected with respective lentivirus, were plated in 15 mm culture dishes, following which the proteins were cross-linked with DNA using 1% formaldehyde. Cross-linking was then stopped using a glycine solution. Sonication was utilised to shear the chromatin to an average fragment size of 500 bp, following which fragmented chromatin was used in the subsequent reaction. DNA was immunoprecipitated with anti-ZFP57 (abcam)^15^ or normal mouse IgG.

Certain primers (namely MEST FW: 5′-GTGGTAGAGCGGCTGGGAG-3′ and RV: 5′-AGAGGAGGTGCCGGGGTG-3′) were used for PCR and qRT-PCR.

### Luciferase reporter assay

A pGL3 reporter containing target regions was transfected into breast cancer cells using a Renilla luciferase vector. Firefly and Renilla luciferase activities were detected using a Dual Luciferase Reporter Assays Kit (Promega, E1910, WI, USA) at 48 h post-transfection according to the manufacturer’s instruction.

TOPFlash/FOPFlash assay was performed in the same way. TOPFlash contains three copies of the TCF-4 binding sites, and FOPFlash (the control reporter) contains mutated TCF-4 binding sites. Renilla luciferase expression plasmid acted as the system control.

### Cell counting kit-8 assay

Cells were seeded at a density of 2000 cells per well in 96-well plates containing a 100 µl cell culture medium. Cell viability was measured using cell cCounting kit-8 (CCK-8) (Beyotime, Shanghai, China) at the time point after transfection, according to the manufacturer’s protocol. Cell proliferation was assessed through measurement of the optical density using an auto-microplate reader.

### 5-Ethynyl-2′-deoxyuridine (EdU) assay

Cells (1 × 10^4^/ml) were seeded into a 24-well plate containing a 2 ml cell culture medium for 24 h. Cell staining was performed using an EdU kit (RiboBio, Guangzhou, China), and images were acquired using fluorescence microscopy (Nikon, Japan).

### Cell cycle analysis by flow cytometry

Cells were collected and washed carefully with PBS and fixed with 75% ethanol at −20 °C. After 24 h fixation, the cells were washed with PBS again and stained with propidium iodide using the cell cycle analysis kit (MultiSciences, Hangzhou, China) for 15 min. Cell cycle features were analysed using a FACScan flow cytometer (BD Bioscience, Franklin Lakes, NJ, USA).

### Subcutaneous xenograft models in vivo

SUM1315 cells (2 × 10^6^ cells in 0.2 ml phosphate-buffered saline (PBS)) which stably transfected lentivirus were injected subcutaneously into the blank area of the forelimb of 6-week-old female Balb/c nude mice (*n* = 6 per group; Animal Core Facility of Nanjing Medical University, Nanjing, China). The tumour diameter in the nude mice was measured every 3 days for 3−4 weeks. After 28 days, all mice were sacrificed and the tumours’ weight and size were measured. The volume of tumour was calculated by using the following formula: volume = (width2 × length)/2. The experiment was approved by the Nanjing Medical University Institutional Animal Care and Use Committee.

### 5-aza-2′-deoxycytidine (5-AzaDc, MCE, USA) treatment

MCF-7 cells were cultured in 10 cm dishes and exposed to 0, 1, 2 and 10 μM of 5-AzaDc at 50−60% confluence for 72 h with a daily medium change.

### DNA extraction and methylation-specific PCR

Genomic DNA from cell lines and tissue specimens was extracted using a TIANamp Genomic DNA Kit (Tiangen, Beijing, China). Extracted DNA was converted via an EpiTect Bisulfite Kit (Qiagen) according to the manufacturer’s instructions. To analyse methylation of MEST DNA, methylation-specific PCR (MS-PCR) was performed using an Epitect MSP kit (Qiagen). The PCR products were analysed using DNA gel electrophoresis. The primer sets are listed in Supplementary file 1 (Part. 2).

### Statistical analysis

Statistical analysis was performed with SPSS 20.0 (Chicago, IL, USA) and Graphpad Prism 6.0. The data were presented as mean ± SD of three independent experiments. The ANOVA with a post hoc test (Dunnett’s multiple comparisons test) for studies with multiple (>2) groups comparisons and Student’s *t* test was used for studies with two groups comparisons. Furthermore, the Pearson correlation was employed to analyse the correlation of expression between ZFP57 and MEST. *χ*^2^ tests were used to analyse the correlation of ZFP57, MEST and the clinical pathological parameters respectively. *P* < 0.05 was considered to be statistically significant. ‘*’ indicates ‘*P* < 0.05’, ‘**’ indicates ‘*P* < 0.01’ and ‘***’ indicates ‘*P* < 0.001’ in all figures.

## Results

### ZFP57 is down-regulated in human breast cancer tissues and cell lines

**Fig. 1:**
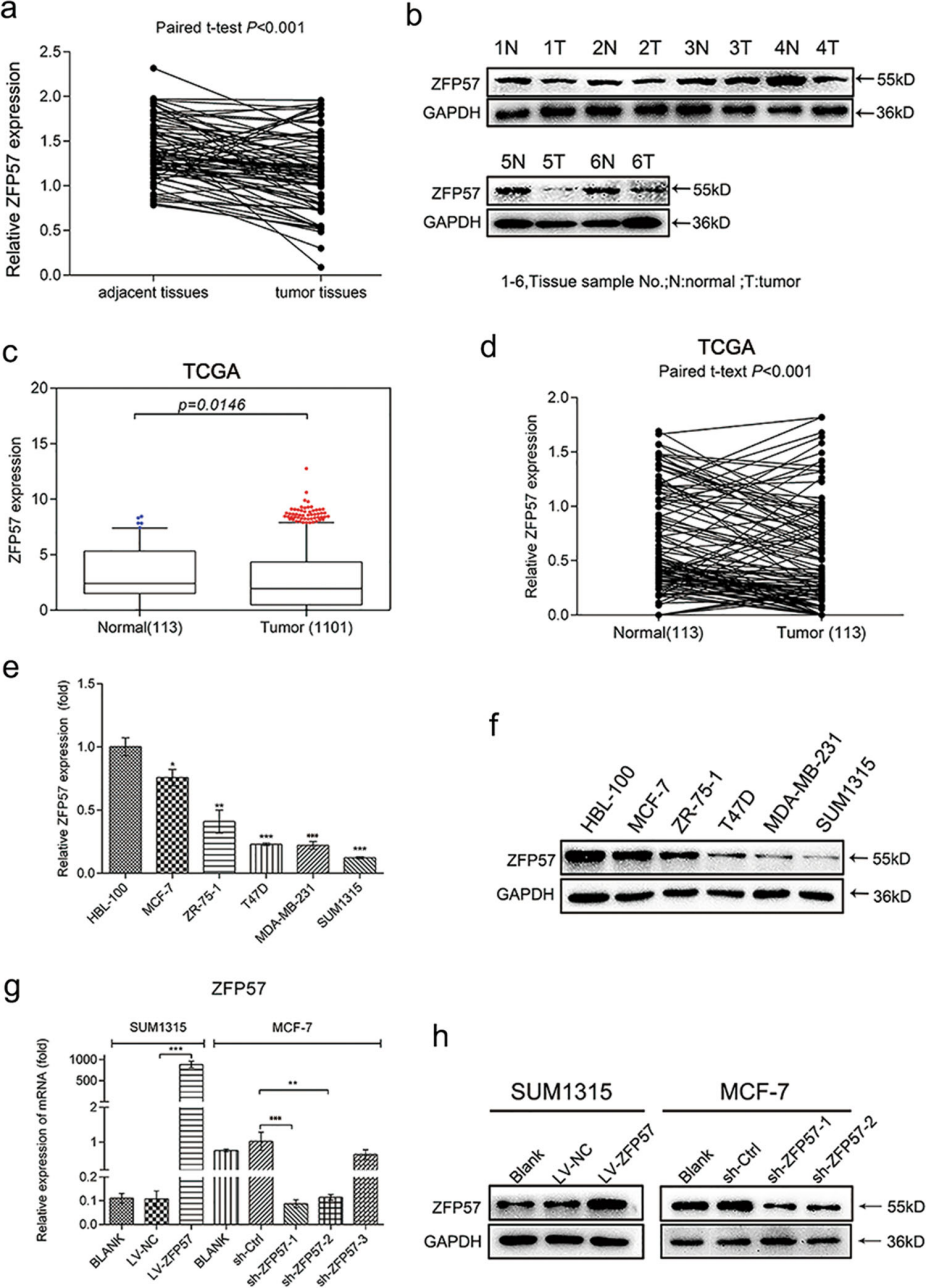
Expression of ZFP57 in breast cancer tissues, cell lines and transfected cells. **a**, **b** Expression of ZFP57 in 80 pairs of human breast cancer tissues and adjacent normal tissues was detected by qRT-PCR (**a**) and 6 pairs by Western blot (**b**). 1–6, Tissue sample NO.; T, tumour tissue; N, normal tissue. **c**, **d** ZFP57 expression in normal breast samples as compared to breast cancer samples (**c**) or paired breast cancer samples (**d**) from TCGA database. **e**, **f** Expression of ZFP57 in breast cancer cells and HBL-100 was detected by qRT-PCR (**e**) and Western blot (**f**). Data are presented as mean ± SD. *p < 0.05; **p < 0.01; ***p < 0.001. **g**, **h** Expression of ZFP57 in cells transfected with LV-NC, LV-ZFP57, sh-Ctrl, sh-ZFP57-1, sh-ZFP57-2 lentivirus and without lentivirus was detected by qRT-PCR (**g**) and Western blot (**h**). Data are presented as mean ± SD. *p < 0.05; **p < 0.01; ***p < 0.001

ZFP57 expression levels in 80 paired human breast cancer specimens and adjacent normal tissues were analysed using mRNA qRT-PCR. The data showed that ZFP57 was obviously decreased in breast cancer tissues (Fig. 1a). Following this, a Western blot was used to detect the expression of ZFP57 protein in six pairs of tissue samples. The ZFP57 expression in breast cancer tissues was of a lower level than that in adjacent normal tissues (Fig. 1b). Moreover, this result was supported by the data from the independent The Cancer Genome Atlas (TCGA) data (Fig. 1c, d). Further, mRNA qRT-PCR and Western blot were performed to examine ZFP57 expression in non-tumourigenic HBL-100 cells and breast cancer cell lines (MCF-7, ZR-75-1, T47D, MDA-MB-231 and SUM1315). ZFP57 expression was lower in the breast cancer cell lines than it was in HBL-100 cells (Fig. 1e, f). The correlation between ZFP57 expression level and clinical features, including age, tumour size, T grade, lymph node metastasis, receptor status and Ki67 was also studied. Patients were respectively allocated to a high- or low-expression group based on whether the expression level of ZFP57 was higher or lower than the median. Table 1 demonstrates that ZFP57 expression was negatively correlated with tumour size larger than 3 cm and high expression of Ki67, which was analysed using Immunohistochemistry.

**Table 1:** Association between ZFP57 and MEST expression and clinicopathologic features of patients with breast cancer (*n* = 80)

Characteristics	Number	Low ZFP57	High ZFP57	*P* value	Low MEST	High MEST	*P* value
*Age (years)*				0.317			0.133
≤60	58	31	27		32	26	
>60	22	9	13		8	14	
*Tumour size (cm)*				0.000			0.044
≤3	43	10	33		26	17	
>3	37	30	7		14	23	
*Lymph node metastasis*				0.370			0.179
Positive	38	17	21		22	16	
Negative	42	23	19		18	24	
T *grade*				0.330			0.021
I/II	69	33	36		38	31	
III/IV	11	7	4		2	9	
*Receptor status*				0.073			0.076
(ER+ or PR+)/(HER2- or HER2 missing)	46	18	28		27	19	
ER−/PR−/(HER2− or missing)	10	6	4		2	8	
HER2 +	24	16	8		11	13	
*Ki67*				0.000			0.025
Low	40	11	29		25	15	
High	40	29	11		15	25	

*ER* oestrogen receptors, *PR* progesterone receptors, *HER2* human epidermal growth factor receptor-2

### ZFP57 inhibits the proliferation of breast cancer cells

SUM1315 and MCF-7 cells were selected for transfection with ZFP57 or shZFP57 lentivirus constructs based on the previously obtained ZFP57 expression levels in breast cancer cell lines; following this, we discussed the biological functions of ZFP57.

**Fig. 2:**
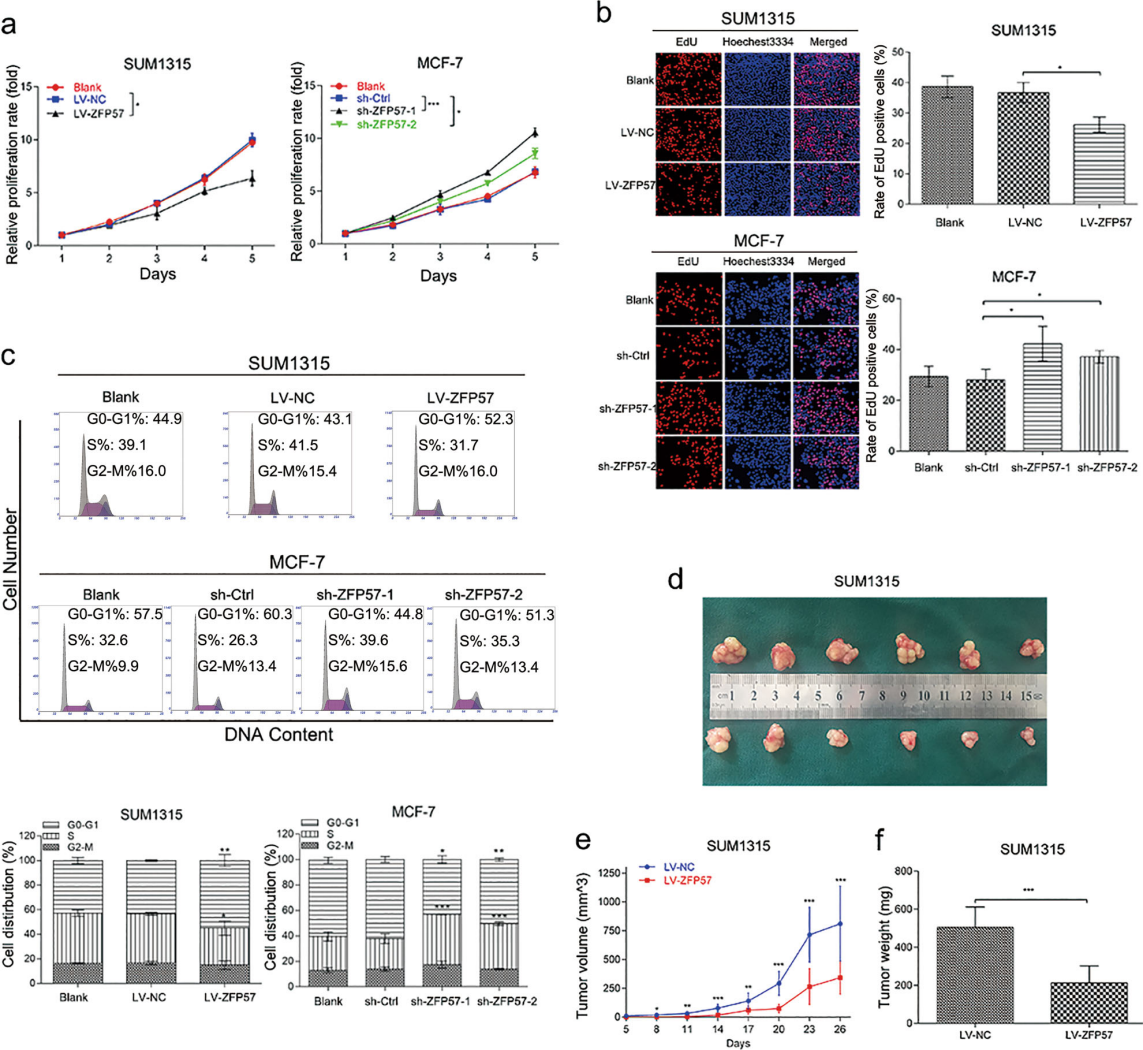
ZFP57 inhibits the proliferation of breast cancer cells. **a**, **b** CCK-8 assay (**a**) and EdU assay (**b**) were used to analyse the proliferation in the ZFP57-overexpressed or -repressed cells as compared with their controls. Data are presented as mean ± SD; *p < 0.05; **p < 0.01; ***p < 0.001. **c** Flow cytometry assay was used to analyse the cell cycle of the ZFP57-overexpressed or -repressed cells as compared with their controls. Data are presented as mean ± SD. *p < 0.05; **p < 0.01; ***p < 0.001. **d** Representative images of the subcutaneous tumours formed in nude mice following injection of ZFP57-overexpressed SUM1315 cells and their respective controls. **e**, **f** Tumour growth curves (**e**) and weight (**f**) are summarised. The average tumour volume and weight are expressed as the mean ± SD of six mice. *p < 0.05; **p < 0.01; ***p < 0.001

For SUM1315, ZFP57 lentivirus was used to improve transfection efficiency, and mRNA qRT-PCR and Western blot were utilised to validate the expression of ZFP57 (Fig. 1g, h). Firstly, CCK-8 assay showed that proliferation of SUM1315 cells was notably suppressed after ZFP57 overexpression for 5 days compared with those transfected with NC vectors (Fig. 2a). As a more specific assessment of proliferation, EdU incorporation assay could confirm the effect on DNA replication. The results appeared to show that ZFP57 obviously reduced the percentage of positive cells compared with the control (Fig. 2b). Flow cytometry was utilised to investigate the relativity of cell-cycle arrest in the subdued proliferation induced by ZFP57 overexpression. SUM1315 cells transfected with ZFP57 exhibited a significant increase in the percentage of cells in the G0/G1 phase, while a decreased percentage was seen in the S phase (Fig. 2c).

Since ZFP57 inhibits cell growth in vitro, we next tested whether ZFP57 could retard the growth of SUM1315 cells in nude mice. Twenty-six days after inoculation, the volumes and weights of tumours infected with SUM1315 cells which overexpressed ZFP57 were smaller than those of the control group (Fig. 2d, f). The tumour growth curves of LV-NC and LV-ZFP57 in nude mice are shown in Fig. 2e.

To further validate the suppression effect of ZFP57 on the growth of breast cancer, we generated a sh-ZFP57 lentivirus and validated the silence effect using mRNA qRT-PCR and Western blot (Fig. 1g, h). CCK8 assay exhibited that the knock-down of ZFP57 in MCF-7, could promote cell growth for 5 days (Fig. 2a). The EdU incorporation assay showed that the positive rate of MCF-7 cells with silent ZFP57 was higher than that of the control cells (Fig. 2b). For MCF-7 cells, knock-down of ZFP57 resulted in a decrease in G0/G1-phase cells. These samples also showed an increase in S-phase cells (Fig. 2c).

Taken together, these findings supported the notion that ZFP57 is involved in the growth of breast cancer cells.

### ZFP57 suppresses the Wnt/β-catenin pathway in breast cancer cells

**Fig. 3:**
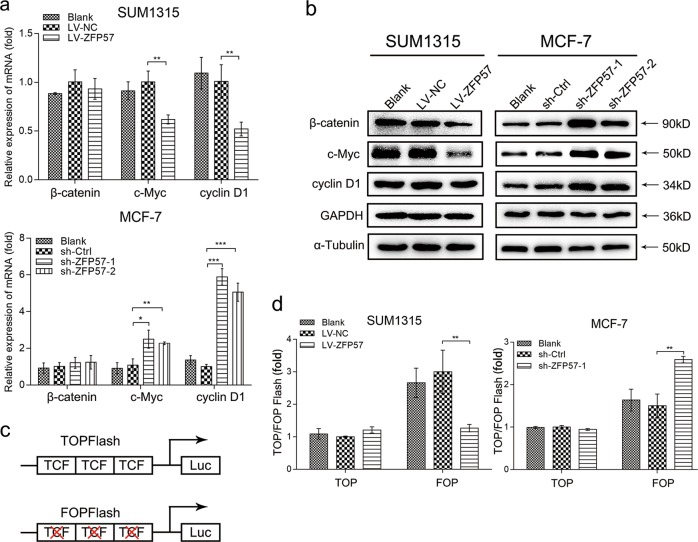
ZFP57 suppresses the Wnt/β-catenin pathway in breast cancer cells. **a** Expression of β-catenin, cyclin D1, c-Myc in the ZFP57-overexpressed or -repressed cells as compared with their controls was detected by qRT-PCR. Data are presented as mean ± SD. *p < 0.05; **p < 0.01; ***p < 0.001. **b** Expression of β-catenin, cyclin D1, c-Myc in the ZFP57-overexpressed or -repressed cells as compared with their controls was detected by Western blot. **c** Schematic diagram of TOPFlash and mutated FOPFlash. **d** The effect of ZFP57 on the Wnt/β-catenin signalling pathway was assessed by dual-luciferase reporter assays (TOPFlash and FOPFlash) in the ZFP57-overexpressed or -repressed cells as compared with their controls. Data are presented as mean ± SD. *p < 0.05; **p < 0.01; ***p < 0.001

To investigate the probability that these effects are mediated partially by the activation situation of the Wnt/β-catenin pathway, the mRNA and protein levels of β-catenin and its downstream target genes (cyclin D1 and c-Myc)^30^ were examined using qRT-PCR and Western blot. The results showed that overexpression of ZFP57 reduced β-catenin protein expression, c-Myc and cyclinD1 mRNA and protein expression in SUM1315 cells, while down-regulation of ZFP57 in MCF-7 cells had the opposite effect (Fig. 3a, b). Interestingly, the mRNA level of β-catenin was not affected after changing the ZFP57 expression (Fig. 3a).

To determine whether the Wnt/β-catenin pathway was affected by ZFP57 in breast cancer cells, a special luciferase reporter assay was utilised. Schematic representation displayed the TOPFlash and TCF site mutant reporter (FOPFlash) (Fig. 3c). The results showed that the ectopic expression of ZFP57 in SUM1315 cells decreased TOPFlash activity, but not FOPFlash activity. Besides this, knock-down expression of ZFP57 in MCF-7 cells increased the TOPFlash activity, but not FOPflash activity (Fig. 3d). These data suggested that ZFP57 could suppress the activity of Wnt/β-catenin pathway.

These data supported the hypothesis that ZFP57 could reduce the proliferation in breast cancer cells by down-regulating the protein level of β-catenin of the Wnt/β-catenin signalling pathway.

### Correlation between ZFP57 and MEST expression in breast cancer

RNA-seq of LV-ZFP57 and LV-NC SUM1315 cells was performed, and the data showed that MEST was down-regulated (>1.5 folds, *P* = 0.017), while there were another 64 differentially expressed RNA sequences in LV-ZFP57 SUM1315 cells (Supplementary file 2).

**Fig. 4:**
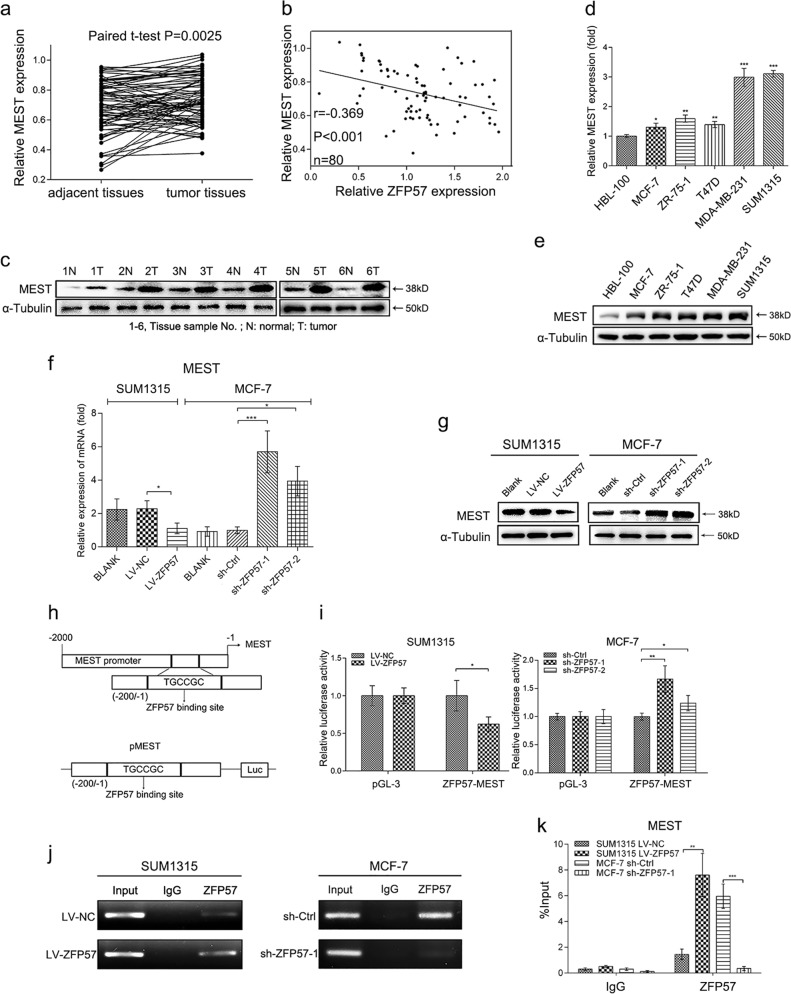
Correlation between ZFP57 and MEST expression in breast cancer. **a**, **c** Expression of MEST in 80 pairs of human breast cancer tissues and adjacent normal tissues was detected by qRT-PCR (**a**) and 6 pairs by Western blot (**c**). 1–6, Tissue sample NO.; T, tumour tissue; N, normal tissue. **b** Negative correlation between the expression of ZFP57 and MEST in breast cancer specimens. **d**, **e** Expression of MEST in breast cancer cells and HBL-100 was detected by qRT-PCR (**d**) and Western blot (**e**). Data are presented as mean ± SD. *p < 0.05; **p < 0.01; ***p < 0.001. **f**, **g** Expression of MEST in the ZFP57-overexpressed or -repressed cells as compared with their controls was detected by qRT-PCR (**f**) and Western blot (**g**). Data are presented as mean ± SD. *p < 0.05; **p < 0.01; ***p < 0.001. **h** Schematic diagram exhibiting potential ZFP57 binding sites elements in promoter region of MEST gene, and schematic representation of the luciferase plasmid with ZFP57 binding sites in promoter region of MEST gene (pMEST). **i** Luciferase assays demonstrating the luciferase activity of pMEST in ZFP57-overexpressed or -repressed cells as compared with their controls. Each error bar indicates the variation between the means of three independent experiments. Data are presented as mean ± SD. *p < 0.05; **p < 0.01; ***p < 0.001. **j**, **k** ChIP analysis was performed in the ZFP57-overexpressed or -repressed cells as compared with their controls. The precipitated chromatin DNA was analysed by conventional PCR (**j**), and qRT-PCR (**k**). Data are presented as mean ± SD. *p < 0.05; **p < 0.01; ***p < 0.001

MEST expression levels in both breast cancer tissues and cell lines were detected. As shown by our results, expression levels of MEST were investigated in breast cancer tissues using mRNA qRT-PCR and Western blot; indeed, MEST was found to be significantly up-regulated in breast cancer tissues as compared with adjacent normal tissues (Fig. 4a, c). These results also exhibited that the mRNA expression levels of ZFP57 had a negative correlation with MEST expression (Fig. 4b). Further, the correlation between expression and clinical features was analysed, and indicated that a higher expression level of MEST was associated with greater tumour size and higher Ki67 expression (Table 1). Moreover, the analysis using mRNA qRT-PCR and Western blot revealed that MEST expression was up-regulated in breast cancer cell lines when compared with HBL-100 cells (Fig. 4d, e).

### MEST is a downstream gene of ZFP57 in breast cancer cells

The good correlation between ZFP57 and MEST expression in tissues led us to further explore the possibility that ZFP57 regulates MEST expression in breast cancer cells. MEST mRNA and protein levels decreased in SUM1315 cells transfected with LV-ZFP57, while both levels increased in MCF-7 cells transfected with sh-ZFP57 lentivirus compared with the negative control, respectively (Fig. 4f, g). Conversely, neither the overexpression nor the knock-down of MEST affected ZFP57 mRNA and protein levels (Fig. 7b, f). Taken together, these results suggested that ZFP57 regulates MEST expression at the transcriptional level and not vice versa.

The TGCCGC hexanucleotide motif is the ZFP57 binding sites^15^. Following this, we noted that the MEST promoter region contained the binding sites (5′-TGCCGC-3′), and that MEST promoter-luciferase constructs (−200 to −1) were designed based on the location of the binding sites (Fig. 4h). Luciferase activity assays showed that luciferase activity was obviously decreased when excess of ZFP57 occurred in SUM1315 cells, while it was increased when ZFP57 was reduced in MCF-7 cells (Fig. 4i). Both the conventional ChIP and qPCR-ChIP analysis showed that ZFP57 overexpression in SUM1315 cells was enhanced, and ZFP57 knock-down in MCF-7 cells reduced ZFP57 levels on the MEST promoter (Fig. 4j, k).

These results indicated that ZFP57 could bind to the promoter region of the MEST in breast cancer cells and then negatively regulate MEST expression.

### ZFP57 in breast cancer cells regulates MEST promoter methylation

**Fig. 5:**
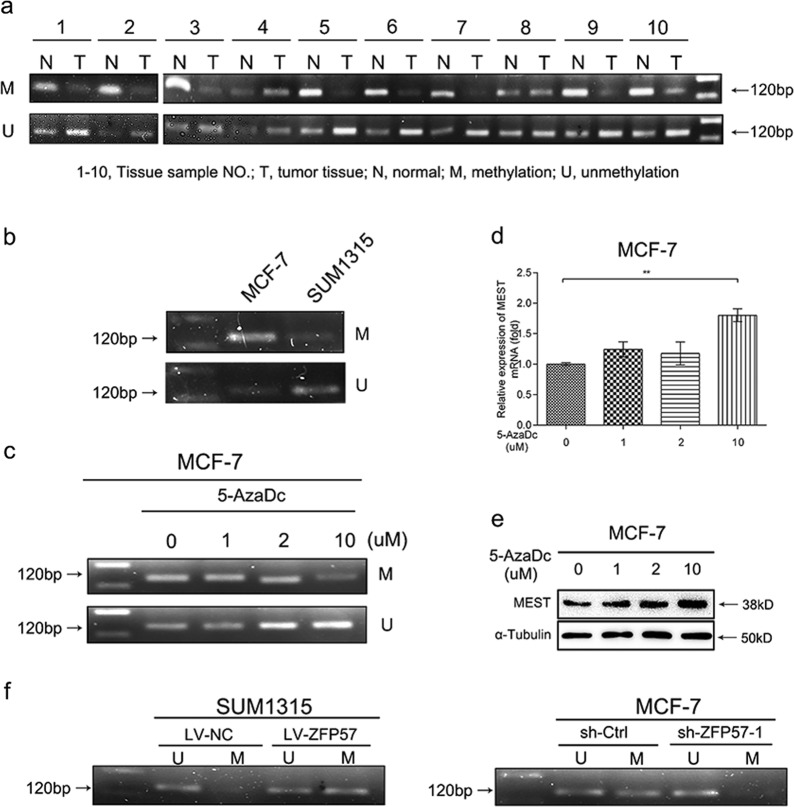
Methylation levels of MEST promoter region in breast cancer tissues and cells, which could be regulated by ZFP57. **a** Representative MS-PCR analysis detected methylation levels of MEST promoter region in ten pairs of human breast cancer tissues and adjacent normal tissues. 1–10, Tissue sample NO.; T, tumour tissue; N, normal tissue; M, methylation; U, unmethylation. **b** Representative MS-PCR analysis detected methylation levels of MEST promoter region in breast cancer (MCF-7 and SUM1315) cells. **c**–**e** Representative MS-PCR analysis (**c**) of MEST, qRT-PCR (**d**) and Western blot (**e**) of MEST expression MCF-7 with or without 5-AzaDc treatment. Data are presented as mean ± SD. **p < 0.01. **f** Representative MS-PCR analysis of MEST in the ZFP57-overexpressed or -repressed cells as compared with their controls

Ten pairs of tissue samples (breast cancer and normal) were randomly distributed for MS-PCR, and 8/10 (80%) of the breast cancer tissue samples had lower methylation levels of MEST promoter versus normal adjacent tissues (Fig. 5a). For breast cancer cells, MCF-7 cells showed higher MEST methylation levels than SUM1315 cells (Fig. 5b). The MCF-7 cells were then treated with different concentrations of the DNA methyltransferase inhibitor (5-AzaDc). MS-PCR showed that the level of methylation obviously decreased after 5-AzaDc treatment, while the level of unmethylation increased (Fig. 5c). Analysis of mRNA qPCR and Western blot showed a dose-dependent restoration of MEST expression after demethylation treatment with 5-AzaDc (Fig. 5d, e). These results suggested that DNA methylation is involved in MEST inactivation in breast cancer.

To confirm the relationship between the ZFP57 expression and DNA methylation levels of MEST, MS-PCR of SUM1315 (LV-NC, LV-ZFP57) cells and MCF-7 (sh-Ctrl, sh-ZFP57) cells was performed. Overexpression of ZFP57 in SUM1315 cells increased MEST methylation levels, while suppression of ZFP57 in MCF-7 cells decreased the levels (Fig. 5f).

Collectively, these data suggested that ZFP57 negatively regulates MEST expression, in part, through maintaining its promoter methylation.

### MEST is the functional target of ZFP57 in breast cancer cells

**Fig. 6:**
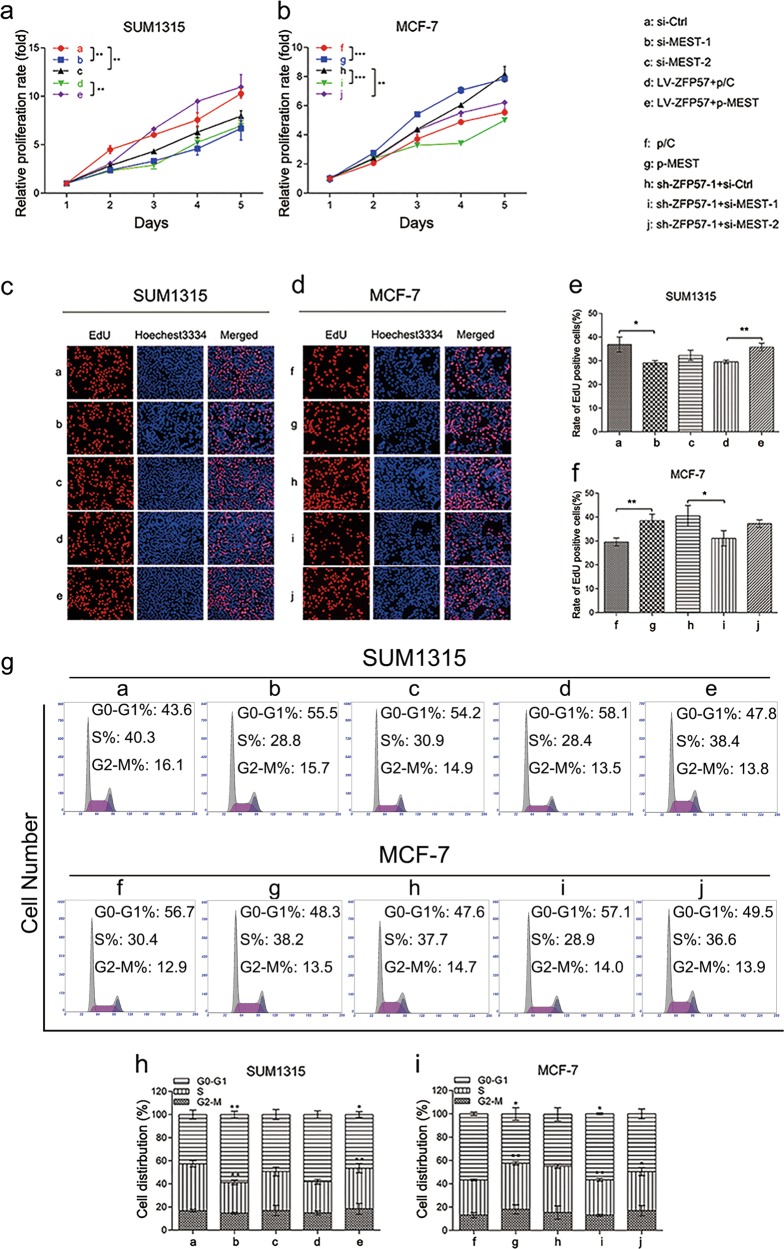
MEST is the functional target of ZFP57 in breast cancer cells. **a**, **b** CCK-8 assays were used to analyse the proliferation of SUM1315 and ZFP57-overexpressed SUM1315 (**a**) or MCF-7 and ZFP57-repressed MCF-7 (**b**) cells, SUM1315 or ZFP57-repressed MCF-7 cells transfected with si-MEST or si-Ctrl and ZFP57-overexpressed SUM1315 or MCF-7 cells transfected with MEST overexpression plasmids or with vector plasmids. Data are presented as mean ± SD. *p < 0.05; **p < 0.01; ***p < 0.001. **c**, **d** The EdU assay. **e**, **f** The data of the EdU assay came from at least three independent experiments. Data are presented as mean ± SD. *p < 0.05; **p < 0.01; ***p < 0.001. **g** Flow cytometry assay was used to analyse the cell cycle. **h**, **i** Flow cytometry assay data came from at least three independent experiments. Data are presented as mean ± SD. *p < 0.05; **p < 0.01; ***p < 0.001. **a** si-Ctrl, **b** si-MEST-1, **c** si-MEST-2, **d** LV-ZFP57 + p/C, **e** LV-ZFP57 + p-MEST, **f** p/C, **g** p-MEST, **h** sh-ZFP57-1 + si-Ctrl, **i** sh-ZFP57-1 + si-MEST-1 and **j** sh-ZFP57-1 + si-MEST-2

To further verify whether MEST mediated the effects of ZFP57 in breast cancer, MEST was down-regulated via transfection with MEST siRNA in SUM1315 cells and up-regulated via transfection with MEST plasmids in MCF-7 cells. MEST knock-down inhibited SUM1315 cell proliferation, which was consistent with the effects of ZFP57 up-regulation (Fig. 6a, c, e, g, h). Upgrading of MEST promoted MCF-7 cell proliferation, which was similar to the effects of ZFP57 down-regulation (Fig. 6b, d, f, g, i).

Moreover, whether the function of MEST could neutralise the effects of ZFP57 overexpression was researched. MEST was down-regulated via transfection with ZFP57 lentivirus, and elevated via transfection with MEST plasmids in SUM1315 cells. The results demonstrated that the suppression of proliferation affected by ZFP57 rising was effectively reversed by ectopic MEST expression (Fig. 6a, c, e, g, h). Similarly, MEST silence in MCF-7 cells which were transfected with sh-ZFP57 lentivirus counteracted the effects of ZFP57 knock-down (Fig. 6b, d, f, g, i).

**Fig. 7:**
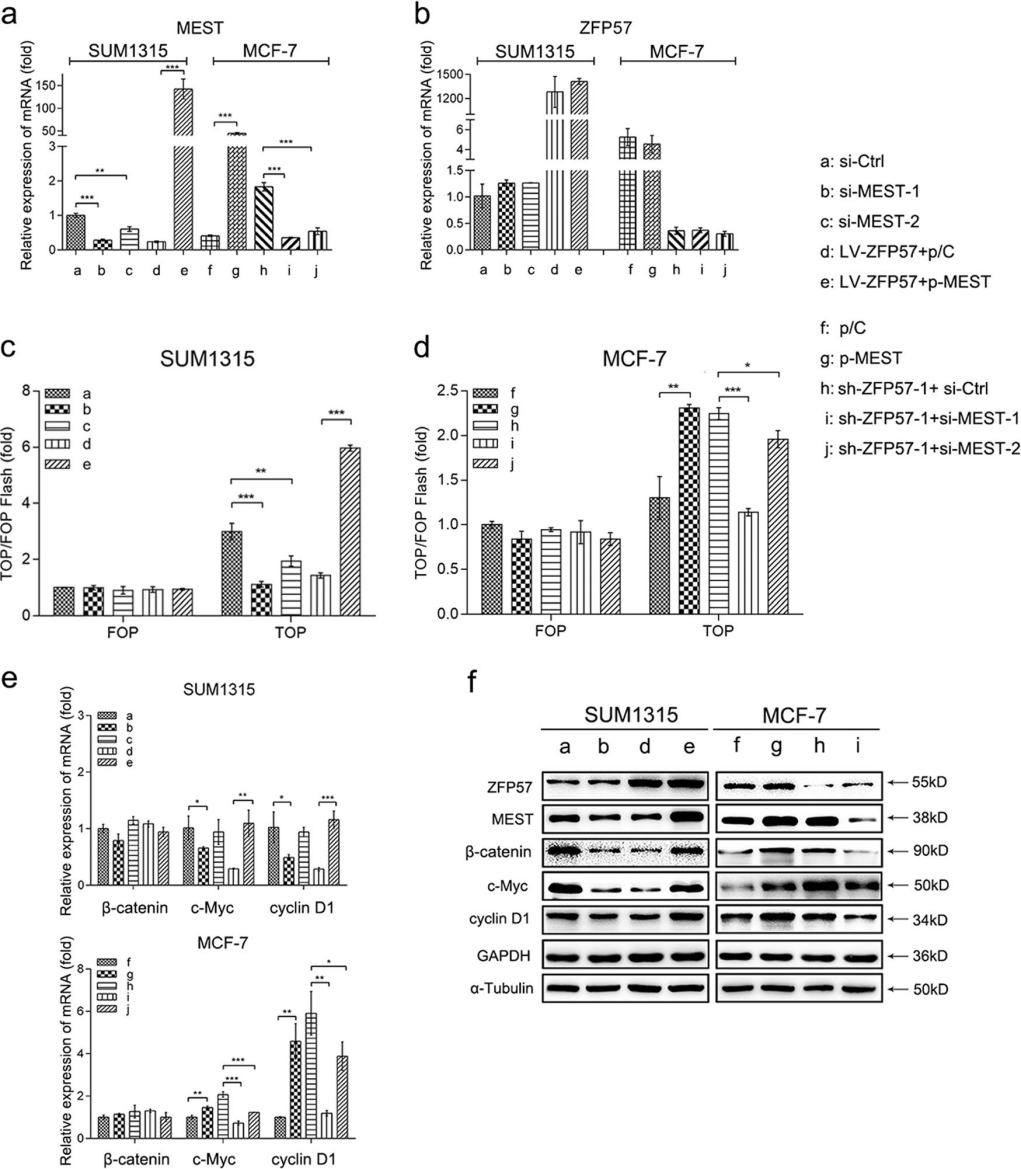
ZFP57 represses the Wnt/β-catenin pathway by down-regulation of MEST. **a**, **b** Expression of MEST (**a**) and ZFP57 (**b**) of SUM1315, ZFP57-overexpressed SUM1315 or MCF-7, ZFP57-repressed MCF-7 cells, SUM1315 or ZFP57-repressed MCF-7 cells transfected with si-MEST or si-Ctrl and ZFP57-overexpressed SUM1315 or MCF-7 cells transfected with MEST overexpression plasmids or with vector plasmids. Data are presented as mean ± SD. *p < 0.05; **p < 0.01; ***p < 0.001. **c**, **d** TOPFlash and mutated FOPFlash assays. Data are presented as mean ± SD. *p < 0.05; **p < 0.01; ***p < 0.001. **e**, **f** Expression of β-catenin, cyclin D1 and c-Myc in SUM1315, MCF-7 and relative cells were detected by qRT-PCR (**e**) and Western blot (**f**). Data are presented as mean ± SD. *p < 0.05; **p < 0.01; ***p < 0.001. **a** si-Ctrl, **b** si-MEST-1, **c** si-MEST-2, **d** LV-ZFP57 + p/C, **e** LV-ZFP57 + p-MEST, **f** p/C, **g** p-MEST, **h** sh-ZFP57-1 + si-Ctrl, **i** sh-ZFP57-1 + si-MEST-1 and **j** sh-ZFP57-1 + si-MEST-2

MEST expression levels in transfected cells were also ascertained (Fig. 7a, f).

Taken together, they illustrated that MEST may be the functional target of ZFP57 in breast cancer.

### ZFP57 represses the Wnt/β-catenin pathway by regulation of MEST

To verify the probability that ZFP57 regulated MEST negatively and then repressed the Wnt/β-catenin pathway, the following assays were performed.

Firstly, the TOPFlash/ FOPFlash luciferase reporter assay was carried out. The data showed that the ectopic expression of MEST in ZFP57 lentivirus-infected SUM1315 cells and MCF-7 cells increased TOPFlash activity (Fig. 7c, d). Besides this, the TOPFlash activity was decreased when MEST expression was silenced in SUM1315 cells and sh-ZFP57 lentivirus-infected MCF-7 cells (Fig. 7c, d). These data suggested that MEST could promote the activity of Wnt/β-catenin pathway.

Following this, the mRNA and protein levels of β-catenin, cyclin D1 and c-Myc were explored. The results appeared to show that, for ZFP57 lentivirus-infected SUM1315 cells and MCF-7 cells, MEST overexpression induced the upgrade of β-catenin, cyclin D1 and c-Myc expression, but not β-catenin mRNA expression (Fig. 7e, f). For SUM1315 cells and sh-ZFP57 lentivirus-infected MCF-7 cells, we also found that the mRNA expression of cyclin D1 and c-Myc and the protein expression of β-catenin, cyclin D1 and c-Myc were reduced when MEST expression was inhibited (Fig. 7e, f).

In summary, it could be suggested that the effects of ZFP57 are mediated via MEST down-regulation and subsequent inactivation of the Wnt/β-catenin pathway.

## Discussion

In recent decades, the incidence of breast cancer among women has risen rapidly worldwide, especially in China^31^. Breast cancer is associated with a variety of genetic variants, including disorders of TSGs and oncogenes^32^. Interestingly, ESCs share many biological properties with cancer cells. In addition, certain regulators and signal transduction pathways of stem cell function have been implicated in cancer pathogenesis^7–10,14^.

As a member of KRAB-ZFPs, ZFP57 was originally identified as an undifferentiated cell-specific gene when cloned from a mouse teratocarcinoma stem cell line, F9^33^. In addition to playing an important role in genome imprinting, ZFP57 can act as the downstream of Stat3, showing self-renewal-specific expression in ESCs^34^. It has also been suggested that ZFP57 serves as a transcriptional repressor in Schwann cells^35^. Since it has been indicated that several KRAB-ZFPs could act as tumour suppressors in multiple types of tumours, such as breast cancer^26–28^, the expression pattern and biological functions of ZFP57 in breast cancer remain to be elucidated. In this study, we primarily confirmed that the expression level of ZFP57 was down-regulated in both breast cancer tissue samples and cell lines compared to adjacent normal tissues and nontumorigenic HBL-100 cells, which is consistent with the TCGA database. Overexpression of ZFP57 inhibited growth of breast cancer cells both in vitro and in vivo significantly. The data indicated that the aberrant expression level of ZFP57 not only correlated with breast cancer development, but could also regulate proliferation activity of breast cancer cells.

Evidence has clarified that KRAB-ZFPs could suppress tumours by inhibiting the Wnt/β-catenin pathway^26^. The Wnt/β-catenin pathway is involved in ESCs’ self-renewal, and its activation is also related to tumorigenesis in a certain set of tissues, including breast tissue^7–10^. Many different mechanisms which could regulate the Wnt/β-catenin pathway are essential for the fine-tuning of signal transduction in physiological and biological processes. The destruction of these constraints occurs in human malignant tumours. The classical Wnt/β-catenin pathway is induced by Wnt ligands that bind to the Frizzled receptor and the LDL receptor-related protein^36–38^. Following this, the degradation of β-catenin is decreased. The stabilisation of β-catenin translocated to the nucleus and interacted with TCF/LEF transcription factors to activate target genes including cyclin D1 and c-Myc, and to promote cancer progression^39^. As an important member of KRAB-ZFPs, it is likely that ZFP57 could inhibit tumour proliferation through the Wnt/β-catenin pathway. Following this, we verified that loss of ZFP57 may stimulate the Wnt/β-catenin pathway by analysing expression levels of β-catenin and its downstream target genes (cyclin D1 and c-Myc). TOPFlash and FOPFlash reporter activity confirmed this.

RNA-seq of ZFP57-overexpressed SUM1315 cells and control group cells was performed, and a total of 65 differentially expressed genes were identified (>1.5 folds, *P* < 0.05), including MEST. The imprinted genes MEST, which is located on chromosome 7 was one of the differentially expressed genes. MEST was ascribed to function as a negative regulator of differentiation while also enhancing cell growth^40,41^. Furthermore, for thyroid cancer, the results showed that MEST was up-regulated and essential for cancer cell survival, and MEST knock-down cells resulted in reduced cell proliferation and G1-phase arrest^42^. These results suggested that MEST may be a potential downstream of ZFP57 in breast cancer. The data showed that the expression level of MEST was negatively correlated with ZFP57 expression. Besides this, the results suggested that elevated or reduced expression of ZFP57 can decrease or increase expression of MEST, respectively in breast cancer cells. Conversely, it was also suggested that change of MEST expression cannot regulate ZFP57 expression. However, there is still a lack of clarity when it comes to the undergoing mechanisms of ZFP57 regulating MEST. A previous study concluded that the imprinting of MEST is frequently lost in invasive breast cancer^43^. The hypothesis that mechanism was loss of imprinting. Loss of imprinting was a well-known mechanism of IGF2 whose expression is also rising in a variety of cancer cells^44,45^. The results of ChIP and dual-luciferase reporter assays showed that ZFP57 could inhibit MEST expression by binding directly to a hexanucleotidic motif in the promoter region of the MEST. Moreover, analysis of MS-PCR appeared to show that the promoter region of the MEST was significantly hypomethylated in breast cancer tissues as opposed to normal adjacent tissues or, rather, MEST expression was regulated by ZFP57 through conserving its DNA methylation in breast cancer cells. With treatment of 5-AzaDc, MEST expression was strengthened in MCF-7 cells, which also supported our hypothesis that demethylation has a vital role in MEST overexpression in breast cancer cells. Following this, data from the CCK-8 assay, EdU incorporation assay and flow cytometry showed that MEST played a key role in the growth of breast cancer cells.

ZFP57 may suppress the Wnt/β-catenin pathway, while we also then found that this effect can be inhibited by the up-regulated expression of MEST in part. TOPFlash and FOPFlash reporter activity confirmed this. Interestingly, no matter whether increasing or decreasing the expression of ZFP57 or MEST, the mRNA levels of β-catenin remained unchanged. Thus, we concluded that MEST could activate the Wnt/β-catenin pathway by conserving β-catenin protein stability rather than promoting gene expression. However, our research did not clarify the specific mechanism by which MEST acts on the Wnt/β-catenin pathway.

In summary, our study demonstrates that the ES-specific transcription factor ZFP57 is a tumour suppressor factor in breast cancer and targets oncogenic MEST directly through regulating the methylation of the MEST promoter region and subsequently suppressing the Wnt/β-catenin pathway. Our discoveries may present a potential diagnostic biomarker and provide a new insight into a novel therapeutic strategy for breast cancer patients.

## Supplementary information

The primers of qRT-PCR and MS-PCR

Data of RNA sequences
