# Supplementary material for: ZFP57 suppress proliferation of breast cancer cells through down-regulation of MEST-mediated Wnt/β-catenin signalling pathway
Source: Cell Death Dis. 2019 Feb 20;10(3):169. doi: 10.1038/s41419-019-1335-5 (PMC6382817; doi:10.1038/s41419-019-1335-5)
Supplement: Supplementary file 1 — The primers of qRT-PCR and MS-PCR [file 41419_2019_1335_MOESM1_ESM.docx]

**Part. 1**

ZFP57 (FW: 5′- CAGAGGGTCCTTTACCAGGAT -3′; RV: 5′- CTCTCAGACTGGGATGTTGTTC -3′);

MEST (FW: 5′- AGTTGTGCTTTTACACGGTTTTC -3′; RV: 5′- CAAGGGCAATCACCCGATGAA -3′);

β-catenin (FW:5′-GGCTACTGTTGGATTGATTCGAA-3′; RV: 5′-GCTGGGTATCCTGATGTGCAC-3′);

c-Myc (FW: 5′-GCGACTCTGAGGAGGAACA-3′; RV: 5′-TGAGGACCAGTGGGCTGT-3′);

cyclin D1 (FW: 5′-AGGAGAACAAACAGATCA-3′; RV: 5′-TAGGACAGGAAGTTGTTG-3′);

GAPDH (FW: 5′-AGAAGGCTGGGGCTCATTTG-3′; RV: 5′-AGGGGCCATCCACAGTCTTC -3′).

**Part. 2**

MEST:

Unmethylated FW:

5′- GGGTTTTAAAAGTTGGTGTTTATTT-3′,

Unmethylated RV:

5′- AACAACTACAACCACTCCAACATA-3′;

Methylated FW:

5′- GGTTTTAAAAGTCGGTGTTTATTC -3′,

Methylated RV:

5′- AACTACAACCACTCCGACGTA-3′.
