# Supplementary material for: ZFP57 suppress proliferation of breast cancer cells through down-regulation of MEST-mediated Wnt/β-catenin signalling pathway
Source: Cell Death Dis. 2019 Feb 20;10(3):169. doi: 10.1038/s41419-019-1335-5 (PMC6382817; doi:10.1038/s41419-019-1335-5)
Supplement: Supplementary file 2 — Data of RNA sequences [file 41419_2019_1335_MOESM2_ESM.docx]

**Additional file 2**

**Figure S1** Heatmap of RNA sequences upregulated or downregulated in SUM1315 cells which transfected with LV-ZFP57 compared with respective controls.


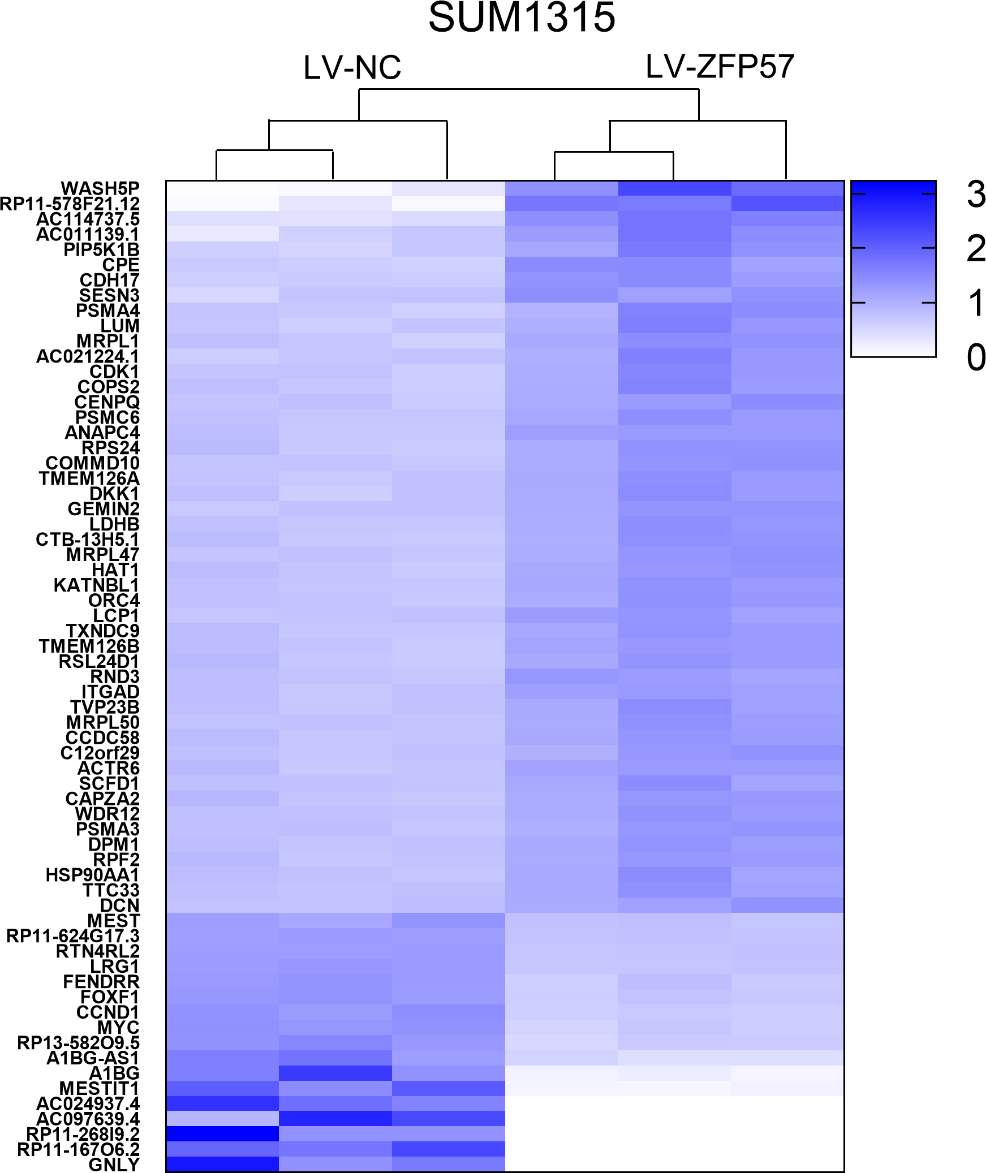


**Table S1** List of RNA sequences data

| ID | Counts | | | | | | FC | logFC | adj.P.Val |
| --- | --- | --- | --- | --- | --- | --- | --- | --- | --- |
|  | LV-NC 1 | LV-NC 2 | LV-NC 3 | LV-ZFP 1 | LV-ZFP 2 | LV-ZFP 3 |  |  |  |
| WASH5P | 1.33333 | 2.66667 | 12 | 53.6667 | 88.6667 | 70.667 | 0.06112 | -4.032 | 0.029536 |
| RP11-578F21.12 | 1 | 6 | 1.6667 | 34 | 32.6667 | 42.667 | 0.07732 | -3.693 | 0.027752 |
| AC114737.5 | 35 | 31 | 39 | 125 | 153 | 141 | 0.25953 | -1.946 | 0.013221 |
| AC011139.1 | 204 | 448 | 529 | 946 | 1326 | 1084 | 0.33799 | -1.565 | 0.036047 |
| PIP5K1B | 92 | 80.5 | 105 | 166 | 252 | 208 | 0.45981 | -1.121 | 0.043033 |
| CPE | 95 | 89 | 81 | 205 | 208 | 165 | 0.47318 | -1.08 | 0.033422 |
| CDH17 | 132.667 | 144 | 140 | 296.667 | 321.333 | 272 | 0.48198 | -1.053 | 0.019589 |
| SESN3 | 80 | 119.667 | 120.33 | 229 | 192 | 222.67 | 0.50437 | -0.987 | 0.049004 |
| PSMA4 | 8367.43 | 7701.43 | 6632.6 | 10764.3 | 17671 | 16030 | 0.53255 | -0.909 | 0.040716 |
| LUM | 2984 | 2478 | 3023 | 4126 | 6623 | 5354 | 0.5482 | -0.867 | 0.037869 |
| MRPL1 | 656 | 598 | 480 | 885 | 1177 | 1133 | 0.55621 | -0.846 | 0.035618 |
| AC021224.1 | 678 | 782 | 828 | 1100 | 1732 | 1415 | 0.56021 | -0.836 | 0.045688 |
| CDK1 | 2715 | 2806.2 | 2359.6 | 3817 | 5620 | 4779.6 | 0.57393 | -0.801 | 0.033422 |
| COPS2 | 2036.33 | 1801 | 1628.3 | 2663.67 | 3928 | 3178 | 0.57851 | -0.79 | 0.037105 |
| CENPQ | 616 | 674 | 540 | 896 | 1065 | 1211 | 0.59384 | -0.752 | 0.037173 |
| PSMC6 | 1741.67 | 1592 | 1593 | 2473 | 3183.33 | 2874 | 0.59495 | -0.749 | 0.018564 |
| ANAPC4 | 383 | 316.5 | 316 | 558.5 | 589 | 593 | 0.59637 | -0.746 | 0.02869 |
| RPS24 | 31106.2 | 24797.8 | 23553 | 37712.2 | 49955.4 | 48900 | 0.59676 | -0.745 | 0.032841 |
| COMMD10 | 470.333 | 481 | 438.67 | 666.667 | 851 | 888 | 0.5969 | -0.744 | 0.035618 |
| TMEM126A | 1502.33 | 1368.67 | 1565.7 | 2160 | 2851 | 2579.3 | 0.60249 | -0.731 | 0.026489 |
| DKK1 | 576 | 453 | 558 | 766 | 1039 | 899 | 0.60334 | -0.729 | 0.045868 |
| GEMIN2 | 347.667 | 399.667 | 399.33 | 545.333 | 699.333 | 705.33 | 0.60635 | -0.722 | 0.040716 |
| LDHB | 17536 | 16009 | 16303 | 23103.5 | 32064 | 29801 | 0.60689 | -0.72 | 0.026489 |
| CTB-13H5.1 | 1252 | 1017 | 1003 | 1497 | 2069 | 1983 | 0.60748 | -0.719 | 0.046771 |
| MRPL47 | 1340.33 | 1419.67 | 1293.7 | 1889 | 2430.33 | 2564.7 | 0.60874 | -0.716 | 0.032841 |
| HAT1 | 2900 | 2544 | 2300 | 3736 | 4543 | 4735 | 0.61062 | -0.712 | 0.028236 |
| KATNBL1 | 789 | 735 | 736 | 1108 | 1406 | 1282 | 0.61325 | -0.705 | 0.027713 |
| ORC4 | 836.2 | 813.6 | 742.2 | 1111 | 1494.6 | 1398.2 | 0.61677 | -0.697 | 0.035978 |
| LCP1 | 801 | 857 | 883 | 1414 | 1504 | 1308.5 | 0.61736 | -0.696 | 0.018564 |
| TXNDC9 | 1434 | 1164 | 1182.7 | 1857.67 | 2276.33 | 2106.7 | 0.62058 | -0.688 | 0.029536 |
| TMEM126B | 2714.33 | 2434 | 2195 | 3713.33 | 4201 | 4081.3 | 0.6263 | -0.675 | 0.018564 |
| RSL24D1 | 4214 | 3466 | 3200 | 5198 | 6414 | 5988 | 0.63197 | -0.662 | 0.033422 |
| RND3 | 363.667 | 342.667 | 318.33 | 593 | 557.667 | 507.33 | 0.63458 | -0.656 | 0.037738 |
| ITGAD | 391 | 328 | 365 | 571 | 608 | 566 | 0.63609 | -0.653 | 0.035169 |
| TVP23B | 1291.5 | 1123 | 1244.5 | 1723.75 | 2307.5 | 1883 | 0.63824 | -0.648 | 0.037411 |
| MRPL50 | 464 | 472 | 451 | 648 | 847 | 745 | 0.63889 | -0.646 | 0.040716 |
| CCDC58 | 921.667 | 782.333 | 802.33 | 1188 | 1452.67 | 1358.7 | 0.64327 | -0.637 | 0.033422 |
| C12orf29 | 1177 | 1076 | 1154 | 1482 | 1942 | 2057 | 0.6434 | -0.636 | 0.047696 |
| ACTR6 | 1520.25 | 1186.75 | 1258.3 | 1975.75 | 2165.25 | 2138.8 | 0.64445 | -0.634 | 0.03074 |
| SCFD1 | 1182 | 1152.67 | 1093.3 | 1634 | 2152.67 | 1686.3 | 0.64707 | -0.628 | 0.037869 |
| CAPZA2 | 1653.5 | 1358 | 1268.3 | 1934.5 | 2425 | 2379 | 0.65039 | -0.621 | 0.049004 |
| WDR12 | 1062 | 1051 | 1009 | 1405 | 1842 | 1705 | 0.65058 | -0.62 | 0.035618 |
| PSMA3 | 3091.33 | 3199 | 2820.7 | 3946.33 | 5181 | 5320.7 | 0.65151 | -0.618 | 0.040716 |
| DPM1 | 2503.5 | 2274.5 | 2379.5 | 3328 | 4177.5 | 3771 | 0.65327 | -0.614 | 0.026489 |
| RPF2 | 1079 | 857 | 941 | 1320 | 1606 | 1568 | 0.65606 | -0.608 | 0.041863 |
| HSP90AA1 | 11172 | 10433 | 9877.5 | 14600 | 19535 | 15255 | 0.65833 | -0.603 | 0.035355 |
| TTC33 | 541.25 | 515.25 | 546.5 | 746.5 | 960.25 | 803 | 0.65872 | -0.602 | 0.045688 |
| DCN | 768.667 | 753.167 | 839.75 | 1079.42 | 1202.17 | 1393.7 | 0.66213 | -0.595 | 0.041725 |
| MEST | 4433.25 | 4042.62 | 4941.3 | 2836.62 | 2944.75 | 2651.9 | 1.62795 | 0.7031 | 0.016986 |
| RP11-624G17.3 | 425 | 450 | 432 | 269 | 277 | 265 | 1.65172 | 0.724 | 0.027713 |
| RTN4RL2 | 1253.67 | 1254.33 | 1299.7 | 739.667 | 746 | 788.67 | 1.71733 | 0.7802 | 0.007166 |
| LRG1 | 1732 | 1809 | 1740 | 969 | 1005 | 1052 | 1.79038 | 0.8403 | 0.002532 |
| FENDRR | 559 | 583 | 552 | 268 | 353 | 289 | 1.91996 | 0.9411 | 0.018564 |
| FOXF1 | 870 | 896 | 825 | 415 | 506 | 455 | 1.93519 | 0.9525 | 0.009356 |
| CCND1 | 15726.5 | 14174 | 16173 | 7040 | 7714 | 7323 | 2.13855 | 1.0966 | 0.000131 |
| MYC | 16002.9 | 15102.1 | 15713 | 6324 | 8101.57 | 7204.9 | 2.23034 | 1.1573 | 0.000224 |
| RP13-582O9.5 | 294 | 323 | 277 | 106.5 | 143.5 | 140.5 | 2.35818 | 1.2377 | 0.018564 |
| A1BG-AS1 | 94.5 | 102 | 70.5 | 32 | 24 | 24 | 3.36932 | 1.7525 | 0.032841 |
| A1BG | 28 | 43 | 24 | 3 | 4 | 2 | 9.4049 | 3.2334 | 0.033422 |
| MESTIT1 | 845 | 595 | 872 | 56 | 44 | 76 | 13.5117 | 3.7561 | 0.000594 |
| AC024937.4 | 10 | 7 | 6 | 0 | 0 | 0 | 16.3978 | 4.0354 | 0.032841 |
| AC097639.4 | 4 | 12 | 10 | 0 | 0 | 0 | 17.2374 | 4.1075 | 0.039426 |
| RP11-268I9.2 | 14 | 6 | 6 | 0 | 0 | 0 | 17.3996 | 4.121 | 0.035618 |
| RP11-167O6.2 | 10 | 9 | 12 | 0 | 0 | 0 | 22.0852 | 4.465 | 0.020852 |
| GNLY | 21 | 10 | 12 | 0 | 0 | 0 | 28.9611 | 4.856 | 0.021028 |
